# Human TMEM2 is not a catalytic hyaluronidase, but a regulator of hyaluronan metabolism *via* HYBID (KIAA1199/CEMIP) and HAS2 expression

**DOI:** 10.1016/j.jbc.2023.104826

**Published:** 2023-05-16

**Authors:** Shinya Sato, Megumi Miyazaki, Shinji Fukuda, Yukiko Mizutani, Yoichi Mizukami, Shigeki Higashiyama, Shintaro Inoue

**Affiliations:** 1 Department of Cosmetic Health Science, Gifu Pharmaceutical University, Gifu, Japan; 2 Department of Biochemistry, School of Dentistry, Aichi Gakuin University, Nagoya, Aichi, Japan; 3 Institute of Gene Research, Yamaguchi University Science Research Center, Ube, Yamaguchi, Japan; 4 Department of Cell Growth and Tumor Regulation, Proteo-Science Center, Ehime University, Toon, Ehime, Japan; 5 Department of Biochemistry and Molecular Genetics, Graduate School of Medicine, Ehime University, Toon, Ehime, Japan; 6 Department of Oncogenesis and Growth Regulation, Osaka International Cancer Institute, Osaka, Japan

**Keywords:** CEMIP, CEMIP2, fibroblast, HAS2, hyaluronan, hyaluronidase, HYBID, interleukin 1, transforming growth factor beta, TMEM2

## Abstract

Cutaneous hyaluronan (HA) is depolymerized to intermediate sizes in the extracellular matrix, and further fragmented in the regional lymph nodes. Previously, we showed that the HA-binding protein involved in HA depolymerization (HYBID), also known as KIAA1199/CEMIP, is responsible for the first step of HA depolymerization. Recently, mouse transmembrane 2 (mTMEM2) with high structural similarity to HYBID was proposed to be a membrane-bound hyaluronidase. However, we showed that the knockdown of human TMEM2 (hTMEM2) conversely promoted HA depolymerization in normal human dermal fibroblasts (NHDFs). Therefore, we examined the HA-degrading activity and function of hTMEM2 using HEK293T cells. We found that human HYBID and mTMEM2, but not hTMEM2, degraded extracellular HA, indicating that hTMEM2 does not function as a catalytic hyaluronidase. Analysis of the HA-degrading activity of chimeric TMEM2 in HEK293T cells suggested the importance of the mouse GG domain. Therefore, we focused on the amino acid residues that are conserved in active mouse and human HYBID and mTMEM2 but are substituted in hTMEM2. The HA-degrading activity of mTMEM2 was abolished when its His248 and Ala303 were simultaneously replaced by the corresponding residues of inactive hTMEM2 (Asn248 and Phe303). In NHDFs, enhancement of hTMEM2 expression by proinflammatory cytokines decreased HYBID expression and increased hyaluronan synthase 2–dependent HA production. The effects of proinflammatory cytokines were abrogated by hTMEM2 knockdown. A decreased HYBID expression by interleukin-1β and transforming growth factor-β was canceled by hTMEM2 knockdown. In conclusion, these results indicate that hTMEM2 is not a catalytic hyaluronidase, but a regulator of HA metabolism.

Hyaluronan (HA), a major component of the extracellular matrix (ECM), is a linear glycosaminoglycan (GAG) consisting of D-glucuronic acid and N-acetyl-D-glucosamine linked *via* alternating β- (1,4) and β- (1,3) glycosidic bonds. It is the largest macromolecular polymer with a molecular mass of several million Daltons (1). Unlike other GAGs, HA does not require a core protein and is abundant in skin and joints, contributing not only to the construction and stability of the ECM but also to various cellular functions such as proliferation, migration, and differentiation (1). Based on its molecular weight, HA has different physiological activities. For example, high-molecular-weight hyaluronan (HMW-HA; molecular mass >1000 kDa) has anti-angiogenic (2), anti-inflammatory, and immunosuppressive effects (3), whereas low-molecular-weight hyaluronan (LMW-HA; molecular mass <100 kDa) has pro-angiogenic (4), proinflammatory (5), and anti-apoptotic effects (6). In addition, HA has a rapid metabolic turnover (turnover rate in the skin is approximately 1 to 1.5 days) (7). Therefore, the balance between HA synthesis and depolymerization must be tightly regulated. In the context of HA synthesis, three HA synthases (HAS) (HAS1, HAS2, and HAS3) have been well characterized (8), and HAS2, which is upregulated by transforming growth factor-β (TGF-β), is predominant in normal human dermal fibroblasts (NHDFs) (9, 10). While members of the hyaluronidase family (HYALs; HYAL1, HYAL2, and HYAL3) have been proposed to degrade HA (11), their involvement in HA degradation and its regulatory mechanisms have not been elucidated, particularly in NHDFs.

To date, we have found that the HA-binding protein involved in HA depolymerization (HYBID), also known as cell migration-inducing and HA-binding protein (CEMIP/KIAA1199), plays an important role in HA-specific depolymerization with an endo-β-N-acetylglucosaminidase activity in human skin and synovial fibroblasts (12). Furthermore, we showed that HYBID depolymerizes HA in early endosomes *via* clathrin-mediated endocytosis and secretes the resulting intermediate-sized fragments. Additionally, its expression is regulated by a range of cytokines (10, 13, 14). Furthermore, HYBID has been shown to be involved in HA degradation not only in human wrinkle formation (15), arthritis (12, 16, 17, 18), and cancer (19, 20, 21) but also in increasing the density of dendritic spines in the mouse dentate gyrus (22).

Mouse transmembrane protein 2 (mTMEM2/mCEMIP2) was recently identified as a novel membrane-bound hyaluronidase (23). The extracellular domain (ECD) of mTMEM2 has 48% amino acid sequence homology to HYBID and a similar domain structure. Using HEK293T cells overexpressing the mTMEM2 gene, Yamamoto *et al*. (23) reported that: (1) mTMEM2 is expressed as a type II transmembrane protein on the cell surface; (2) the recombinant mTMEM2 protein degrades HMW-HA to approximately 5 kDa HA; (3) it has no chondroitin sulfate or dermatan sulfate degrading activity; (4) it is a Ca^2+^ dependent hyaluronidase with an optimum pH of 6 to 7. Studies that have primarily used mTMEM2 knockout (KO) mice have shown that mTMEM2 has functions in systemic hyaluronan catabolism and turnover (24), and neural crest cell development and survival (25).

However, for human-derived TMEM2 (hTMEM2), HA-degrading activity has not been directly assessed although gene and protein expression levels have been analyzed. In human fibroblasts in which hTMEM2 was knocked out by CRISPR/Cas9 to evaluate ER stress sensitivity, hTMEM2 was reported to be involved in longevity associated with mitochondrial damage (26). In this study, phenotypic recovery of hTMEM2-KO was confirmed by the addition of active sheep hyaluronidase or LMW-HA, and the HA-degrading activity of fibroblasts with TMEM2-KO was not directly determined. In another example, using a variety of human-derived tumor cell lines, cell adhesion, and migration were shown to be regulated by hTMEM2 *via* HA degradation of cell focal adhesion sites (27). However, in this report, phenotypic recovery of cells with hTMEM2-KO was evaluated by introducing mTMEM2 instead of hTMEM2, or by adding small HA fragments.

In our previous study using NHDFs, we observed the following: (1) HA degradation was completely suppressed by hHYBID knockdown (KD), however, it increased with hTMEM2-KD and (2) TGF-β, which suppressed HA degradation with decreased hHYBID expression, promoted hTMEM2 expression (12). These results indicate that hHYBID is essential for HA degradation in NHDFs and that hTMEM2 suppresses HA degradation by decreasing HYBID expression. Furthermore, single or prolonged stimulation of NHDFs with proinflammatory cytokines increased the expression levels of HAS2 and TMEM2, suppressed HYBID expression, and resulted in the accumulation of HMW-HA in the medium (13). These results strongly suggest that hTMEM2 does not function as a hyaluronidase, but does so as a regulator of HA metabolism.

In this study, we aimed to investigate whether hTMEM2 has a catalytic HA-degrading activity, using HEK293T cells. Furthermore, we designed chimeric and amino acid residue-replaced mTMEM2 proteins to determine the important domains for the HA-degrading activity. Then, we examined the effects of hTMEM2-KD on the cytokine-induced *HYBID* and *HAS2* gene expressions. Finally, we clarified that hTMEM2 lacks catalytic hyaluronidase activity but is a regulator of HA metabolism that promotes the accumulation of HMW-HA by reducing HYBID-dependent HA depolymerization and increasing HAS2-dependent HA production.

## Results

### Human TMEM2 does not function as a hyaluronidase

**Figure 1:**
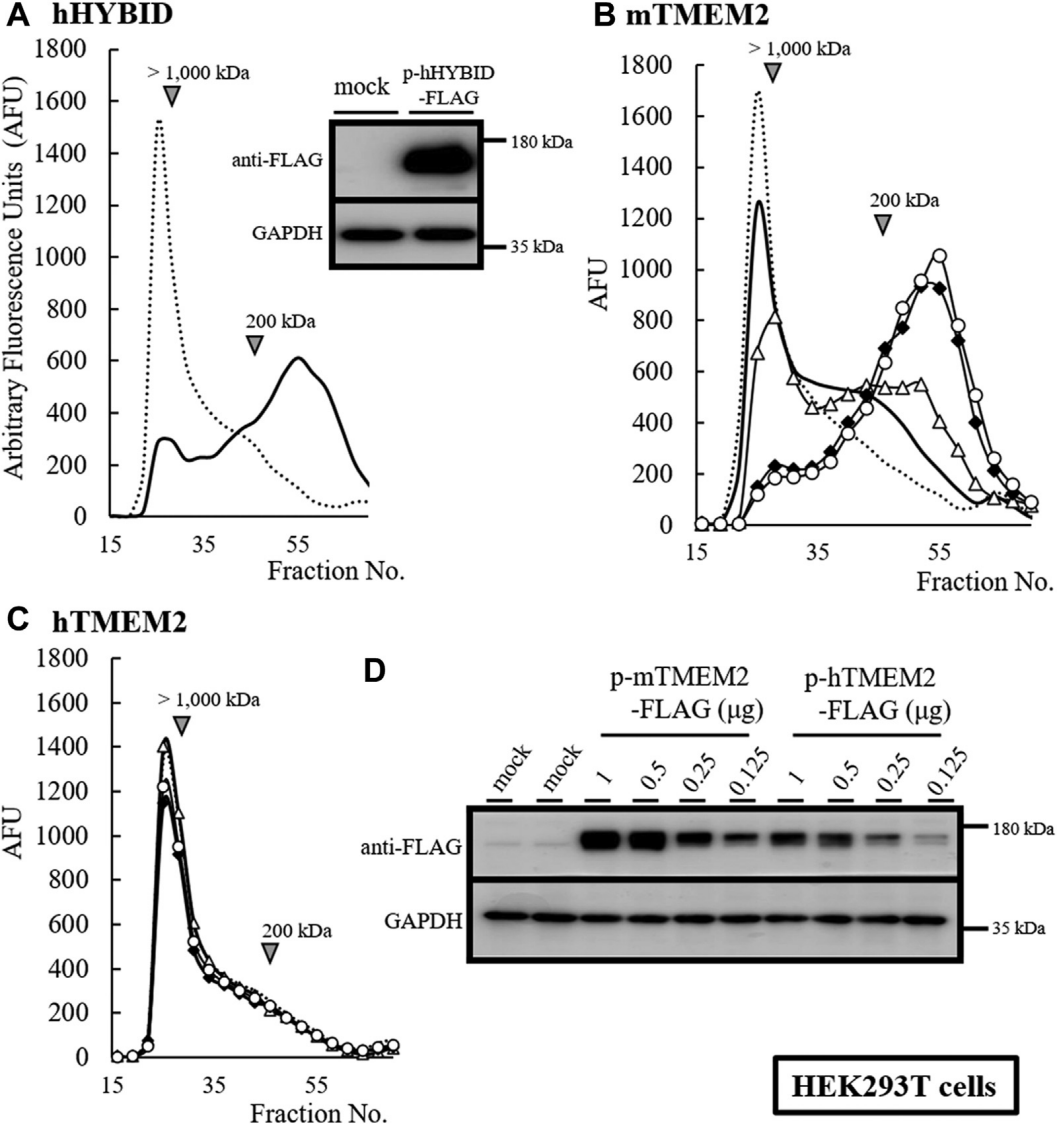
**Human TMEM2 does not depolymerize HMW-HA in HEK293T cells**. Cellular HA depolymerization activity was detected using the mouse or human TMEM2 overexpression system. *A*, in HEK293T cells, the expression of hHYBID (hHYBID-FLAG-pcDNA3.1) acts as the positive control showing HA depolymerization (*solid line*). FITC-labeled HMW-HA was added to the medium and cultured for 48 h. The distribution of FITC-labeled HA was determined by the Sepharose CL-2B column (0.7 × 50 cm). The depolymerized HA was detected under 200 kDa or less (around fraction No. 55). FITC-labeled HMW-HA is a substrate (*dotted line*). *B* and *C*, the dose-dependent reaction of HA depolymerization using the HEK293T cell overexpression system. Mouse (*B*, mTMEM2-FLAG-pcDNA3.1, 0.125 [no mark], 0.25 [*triangle*], 0.5 [*diamond*], and 1.0 [*circle*] μg) and human (*C*, hTMEM2-FLAG-pcDNA3.1, 0.25 [*triangle*], 0.5 [*diamond*], and 1.0 [*circle*] μg) TMEM2-FLAG expression plasmids were transfected into HEK293T cells and the HA depolymerization activity was detected using FITC-labeled HMW-HA. *D*, the protein expression levels of FLAG-tagged TMEM2 in the HEK293T cells were determined *via* immunoblotting. GAPDH was used as the loading control.

**Figure 2:**
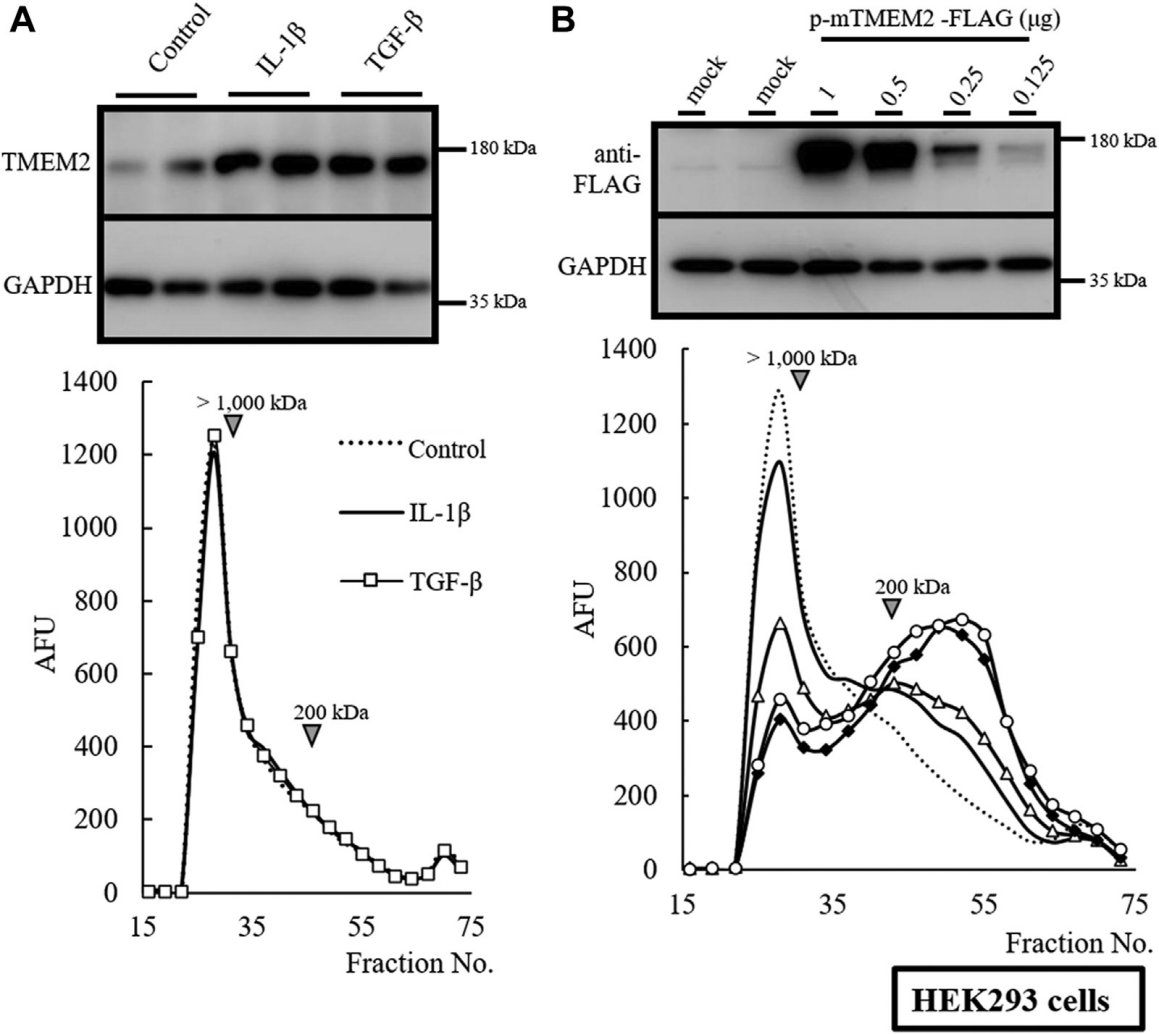
**Endogenous expression of hTMEM2 in HEK293 does not induce HA depolymerization**. *A*, effects of IL-1β and TGF-β on endogenous HA depolymerization system in HEK293 cells. HEK293 cells were stimulated with or without IL-1β (1 ng/ml) and TGF-β (10 ng/ml) for 48 h in HEK293 cells. Endogenous hTMEM2 expression was detected by immunoblotting using an anti-TMEM2 antibody. After stimulation, HEK293 cells were cultured with FITC-labeled HMW-HA for 48 h. For the detection of HA depolymerization, the distribution of FITC-labeled HA was determined by the Sepharose CL-2B column (0.7 × 50 cm). *B*, overexpressed mTMEM2 activity in HEK293 cells. The dose-dependent expression of mTMEM2 protein in HEK293 cells was detected by immunoblotting using an anti-FLAG antibody (mTMEM2-FLAG-pcDNA3.1, 0.125 [no mark], 0.25 [triangle], 0.5 [*diamond*], and 1.0 [circle] μg). FITC-labeled HMW-HA was added to the medium and cultured for 48 h. The HA depolymerization reaction was detected as increasing under 200 kDa or less fractions. The control substrate is shown as a dotted line. GAPDH was used as an immunoblotting loading control.

To verify the HA-degrading activity of hTMEM2, we cultured *mTMEM2*-and *hTMEM2*-transfected HEK293T cells. Neither endogenous hHYBID nor hTMEM2 expression was detectable in the cells. We detected the hHYBID activity in cells that served as the positive control (Fig. 1*A*). FITC-labeled HMW-HA (200–1600 kDa) was depolymerized to intermediate-sized fragments of around 200 kDa or less by hHYBID. Similarly, we detected dose-dependent HA-degrading activity in mTMEM2-expressing HEK293T cells (Fig. 1, *B* and *D*). However, HEK293T cells expressing hTMEM2 protein showed no HA-degrading activity (Fig. 1, *C* and *D*), indicating that hTMEM2 expressed in HEK293T cells lacked HA-degrading activity under conditions where HMW-HA was depolymerized by hHYBID and mTMEM2. To rule out the possibility of lack of activity owing to an unexpected hTMEM2-expression system using HEK293T cells, we examined the HA-degrading activity of endogenously expressed hTMEM2 in HEK293 cells instead of HEK293T cells. Stimulation with interleukin-1β (IL-1β) and TGF-β markedly enhanced the expression of hTMEM2 in HEK293 cells (Fig. 2*A*) as previously observed in NHDFs (13, 14). However, IL-1β- or TGF-β-enhanced expression of hTMEM2 in HEK293 cells did not show any HA-degrading activity. HEK293 cells expressing mTMEM2 could degrade extracellular HA (Fig. 2*B*), strongly indicating that HEK293 cells cannot degrade HA on their own.

### Mouse GG domain and C-terminal tail are essential for HA-degrading activity

**Figure 3:**
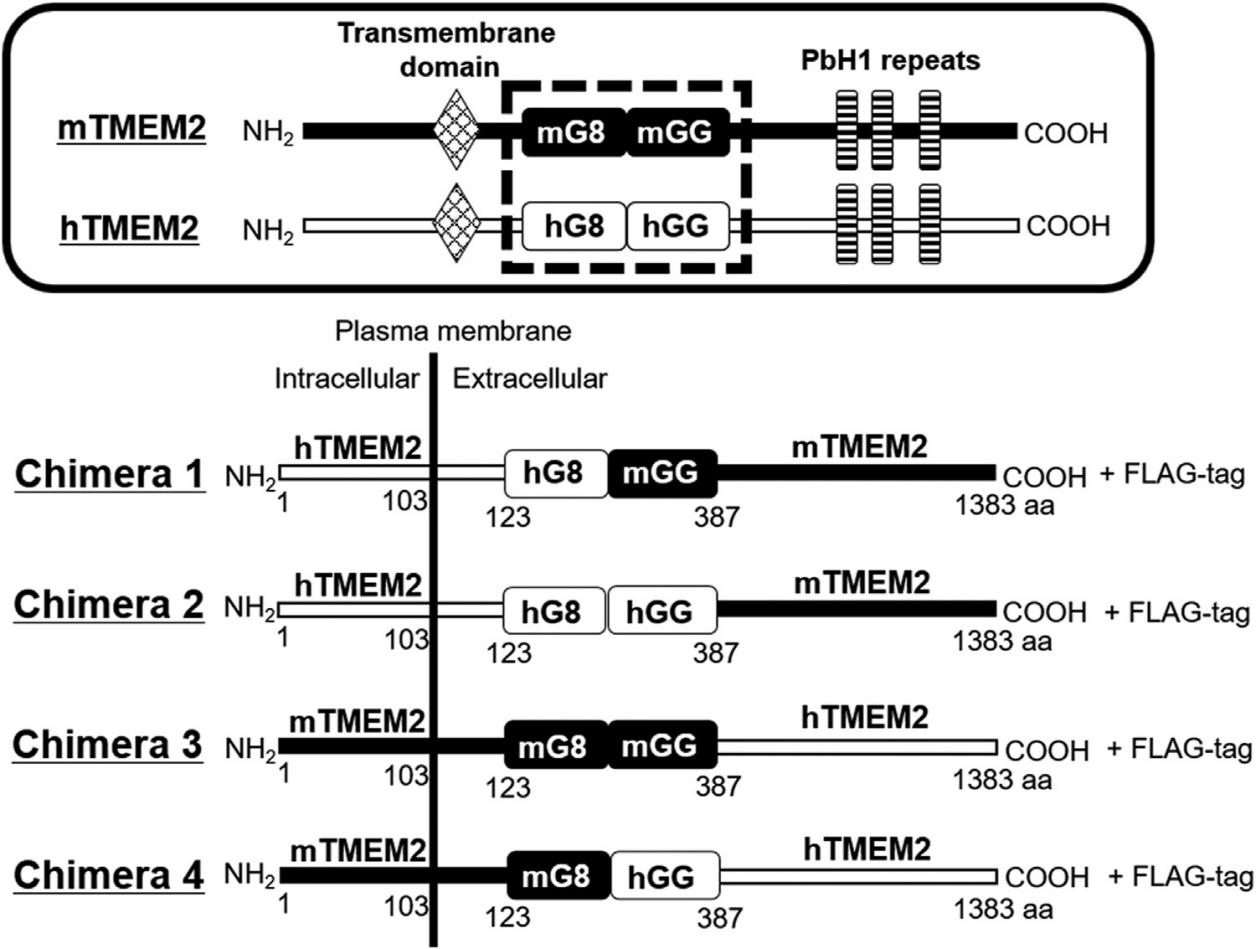
**The design of chimera construction of mouse and human TMEM2.** Chimeric constructs of human and mouse TMEM2 targeting the GG domain. The TMEM2 protein consists of an N-terminal intracellular domain, a transmembrane domain, and a C-terminal extracellular domain containing G8 domain, GG domain, and three PbH1 repeats. Chimera 1 has a human G8 (hG8), mouse GG (mGG) domain, and mouse C-terminal tail. Chimera 2 has hG8, human GG (hGG), and mouse C-terminal tail. Chimera 3 has mouse G8 (mG8), mGG, and human C-terminal tail. Chimera 4 has mG8, hGG, and human C-terminal tail.

**Figure 4:**
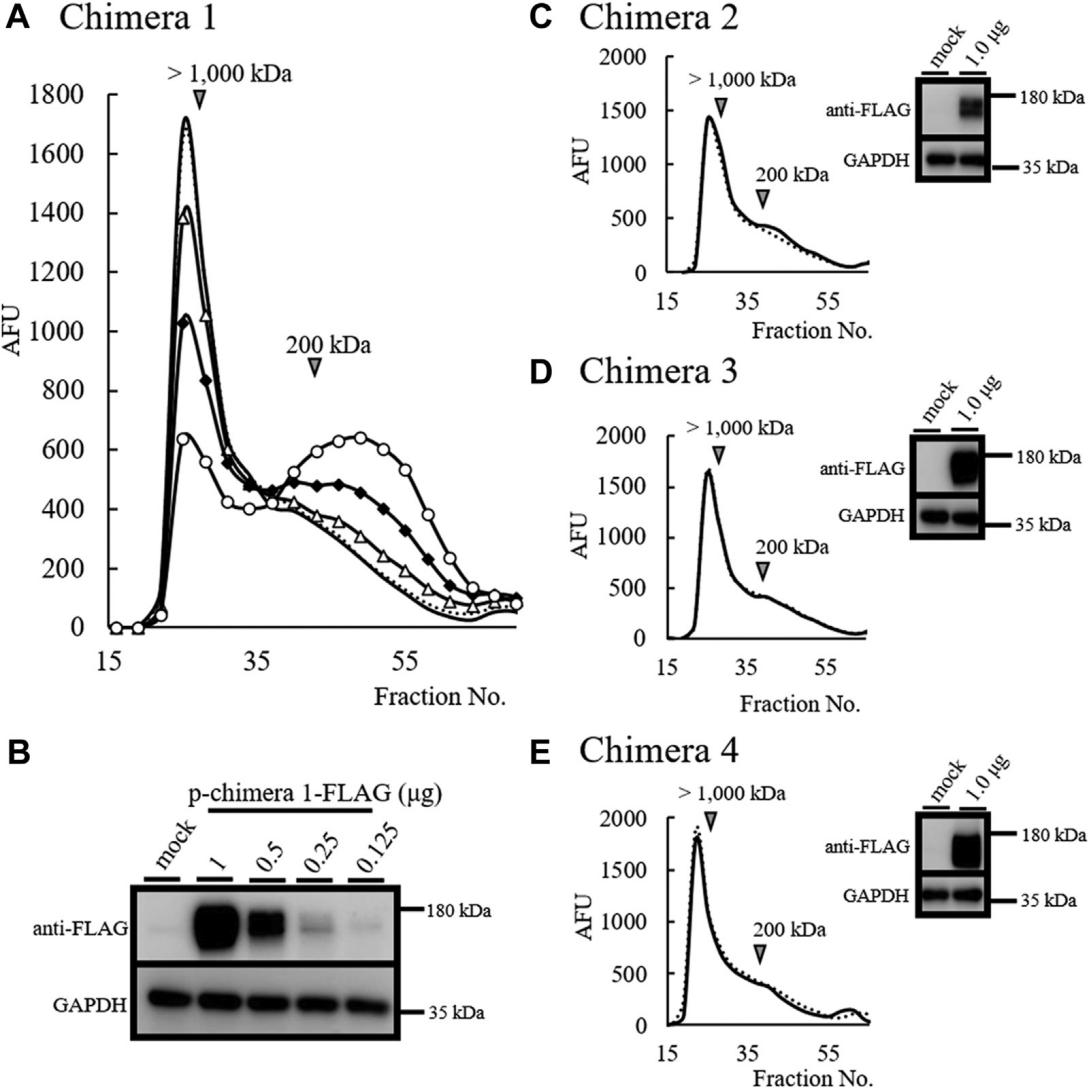
**Mouse GG domain and C-terminal tail are essential for HA depolymerization**. *A*, dose-dependent HA depolymerization reaction of human and mouse chimeric TMEM2-1 (Chimera 1, hG8/mGG) expression in HEK293T cells. After transfection of hmTMEM2 Chimera 1-FLAG-pcDNA3.1 (0.125 [no mark], 0.25 [*triangle*], 0.5 [*diamond*], and 1.0 [*circle*] μg), cellular HA depolymerization activity was detected using FITC-labeled HMW-HA. The active reaction peak was detected by fragments under 200 kDa or less. The control substrate is shown as a *dotted line*. *B*, the expression levels of FLAG-tagged Chimera 1 (hG8/mGG) protein in the HEK293T cells were detected by immunoblotting. *C*–*E*, effects of Chimera 2, 3, and 4 expressions in HEK293T cells on HA depolymerization (*solid line*). The control substrate is shown as a *dotted line*. The expression levels of Chimera 2, 3, and 4 proteins were detected using anti-FLAG antibody immunoblotting. GAPDH was used as an immunoblotting loading control.

Mouse and human TMEM2 have a similar protein structure with approximately 66% and 87% homology (23), on their nucleotide and amino acid sequences, respectively (Fig. 3). Both TMEM2s carry the G8 and GG domains and contain three PbH1 repeats in the C-terminal ECD. To determine the domains essential for the HA-degrading activity of mTMEM2, we prepared chimeric constructs of human and mouse TMEM2. As the GG1 domain of hHYBID and GG domain of mTMEM2 are critical for their enzymic activity (12, 23), we constructed chimeric TMEM2 focusing on the G8 and GG domains, with the mouse and human domains swapped. First, we examined Chimera 1 which has a human N-terminal, human G8 (hG8), and mouse GG (mGG) domains, with a mouse C-terminal tail. HEK293T cells expressing Chimera 1 degraded extracellular HA in a dose-dependent manner (Fig. 4, *A* and *B*). However, no degrading activity was detected in other HEK293T cells expressing Chimera 2 (hG8, hGG, and mouse C-terminal tail), Chimera 3 (mG8, mGG, and human C-terminal tail), and Chimera 4 (mG8, hGG, and human C-terminal tail) (Fig. 4, *C*–*E*). These results indicate that the mouse GG domain and C-terminal tail are essential for HA-degrading activity.

### His248 and Ala303 of mTMEM2 are essential for HA-degrading activity

**Figure 5:**
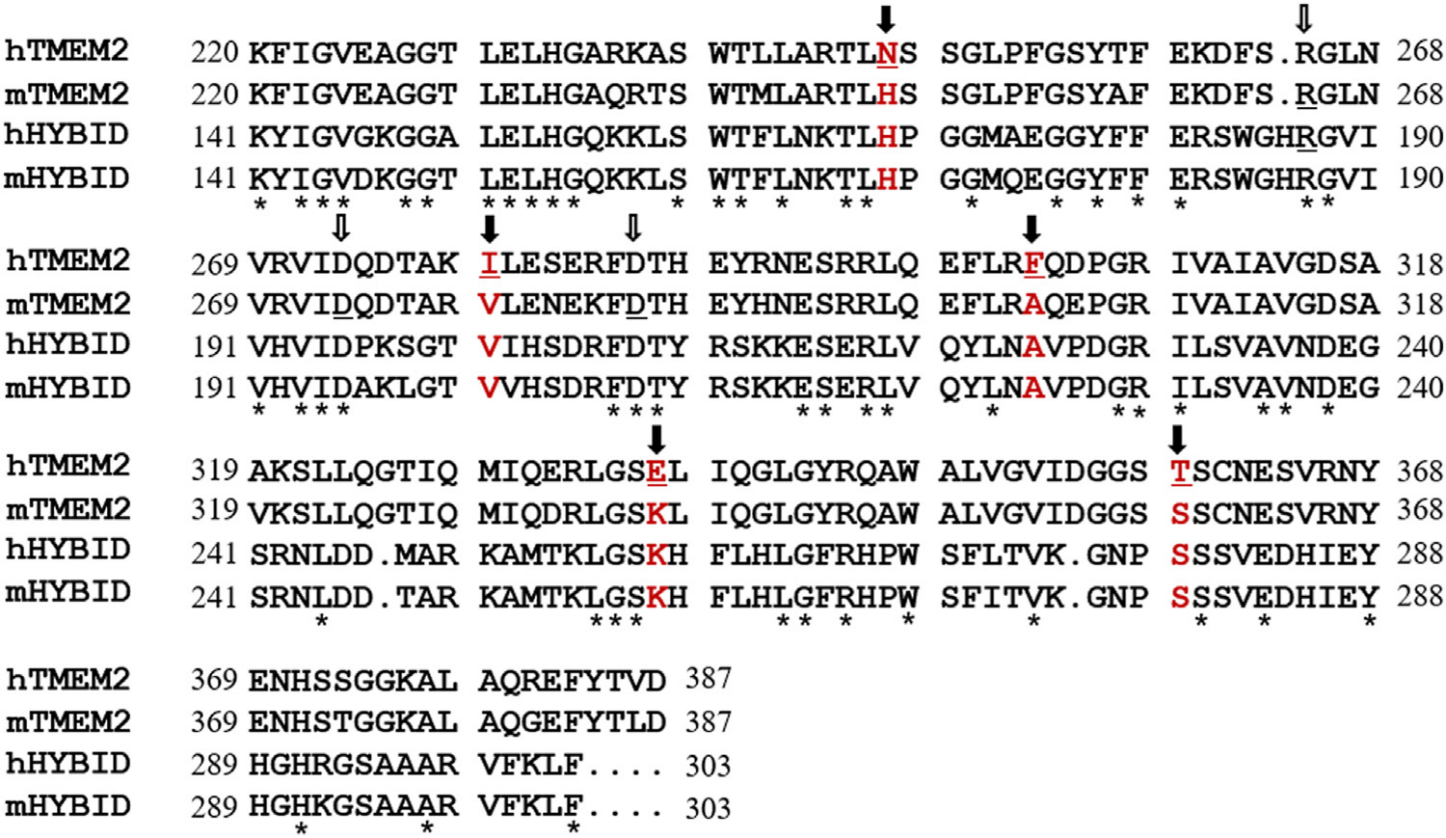
**Sequence alignment of GG domain areas between human and mouse TMEM2 and HYBID**. Sequence alignment of GG domain between hTMEM2 (220–387 aa), mTMEM2 (220–387 aa), hHYBID (141–303 aa), and mHYBID (141–303 aa). Residues indicated by *black arrows* (amino acids that are not conserved in human TMEM2 only) were replaced with human amino acids in the mTMEM2 sequence as follows: H248N, V279I, A303F, K337E, S359T, and H248N/A303F. Amino acids conserved between all of the proteins are indicated by *asterisks*. It has been reported that R187C and R187H in the GG domain of hHYBID (12), and R265C, D273N and D286N in the GG domain of mTMEM2 (23) cause loss of HA-degrading activity (*white arrows*).

**Figure 6:**
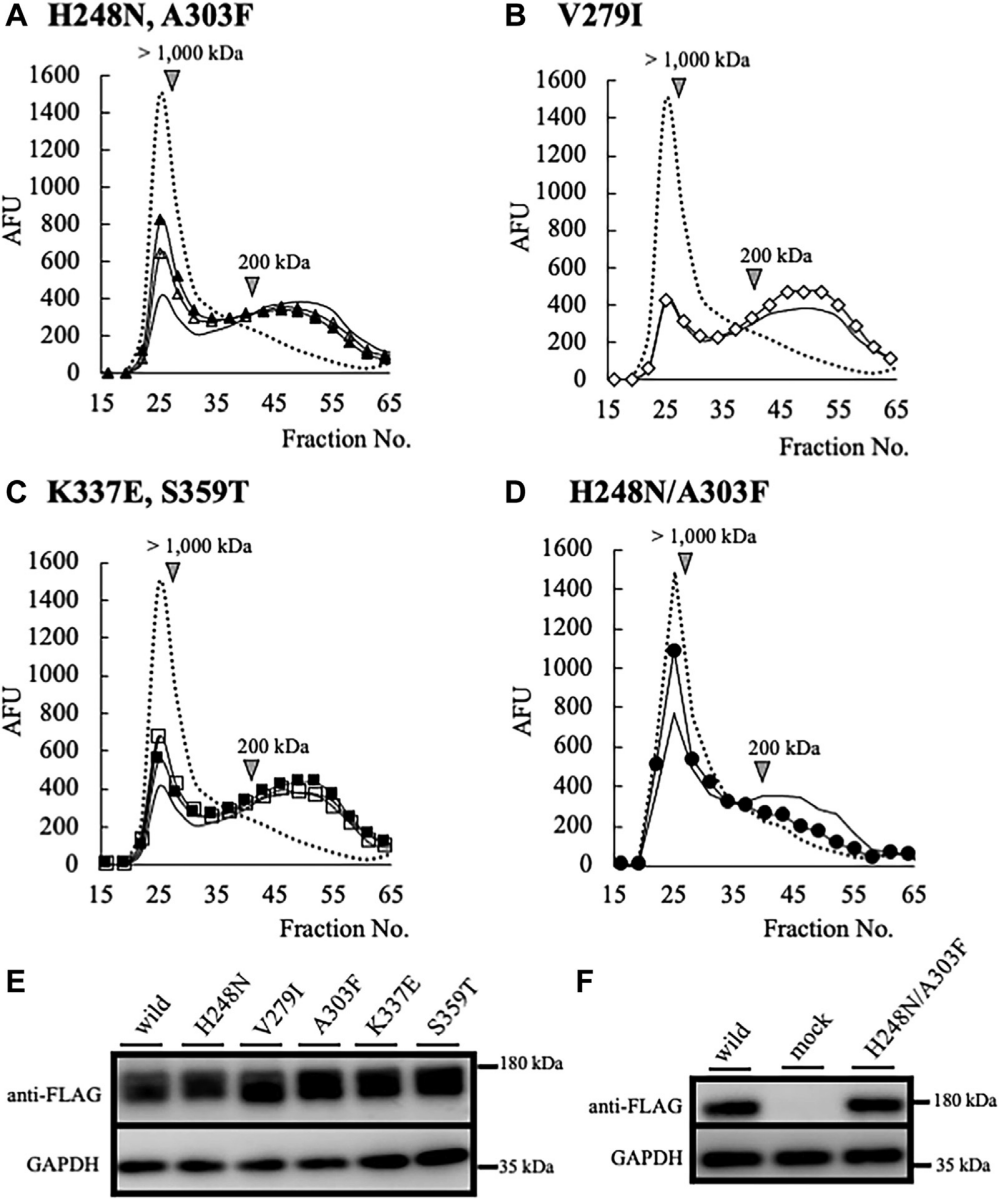
**His 248 and Ala 303 of mTMEM2 are essential for HA depolymerization activity**. *A*–*C*, comparison of HA depolymerization activity between single amino acid replacement proteins. Single replacement constructs (*A*, H248N [*open triangle*] and A303F [*closed triangle*]; *B*, V279I [*open diamond*]; *C*, K337E [*open square*] and S359T [*closed square*]) were transfected into HEK293T cells and incubated with FITC-HMW-HA for cellular HA depolymerization assays. *D*, a double replacement construct (H248N/A303F [*closed circle*]) was transfected into HEK293T cells. FITC-HMW- HA was added to the cells for 48 h and the culture medium was subjected to gel chromatography. Wild-type mTMEM2 represents the positive control (no mark), and negative control is shown as a dotted line. *E* and *F*, mTMEM2 single (*E*) and double (*F*) replacement proteins detected by FLAG-tagged protein expression levels. mTMEM2 (wild) is a positive control and an empty vector (mock) serves as the negative control. GAPDH was used as an immunoblotting loading control.

Figure 5 shows the comparison of amino acid sequences of GG domains between TMEM2 and HYBID. As the GG domain is essential for the HA-degrading activity of mTMEM2, we focused on the amino acid residues in the domain that are conserved in active hHYBID, mHYBID, and mTMEM2, but substituted in hTMEM2. The corresponding residues were located at five positions, 248, 279, 303, 337, and 359, that differ only in the GG domain of hTMEM2 (Fig. 5). In this study, we replaced these five amino acid residues of mTMEM2 with the corresponding hTMEM2 amino acid (H248N, V279I, A303F, K337E, and S359T). The HA-degrading activity of HEK293T cells expressing mTMEM2 containing these five amino acid substitutions is shown in Figure 6. Compared to native mTMEM2, we detected a marked suppression of HA degradation in mTMEM2 with two simultaneous amino acid substitutions (H248N/A303F) (Fig. 6*D*), whereas a single replacement of each amino acid residue resulted in only a slight suppression (Fig. 6*A*). On the other hand, HA degradation was slightly enhanced when V279I was substituted (Fig. 6*B*) and unchanged when K337E and S359T were substituted (Fig. 6*C*). In these experiments, the expression levels of the FLAG-tagged proteins were nearly identical (Fig. 6, *E* and *F*). This finding indicates that His248 and Ala303 in the GG domain are essential for the catalytic HA-degrading activity of mTMEM2.

**Figure 7:**
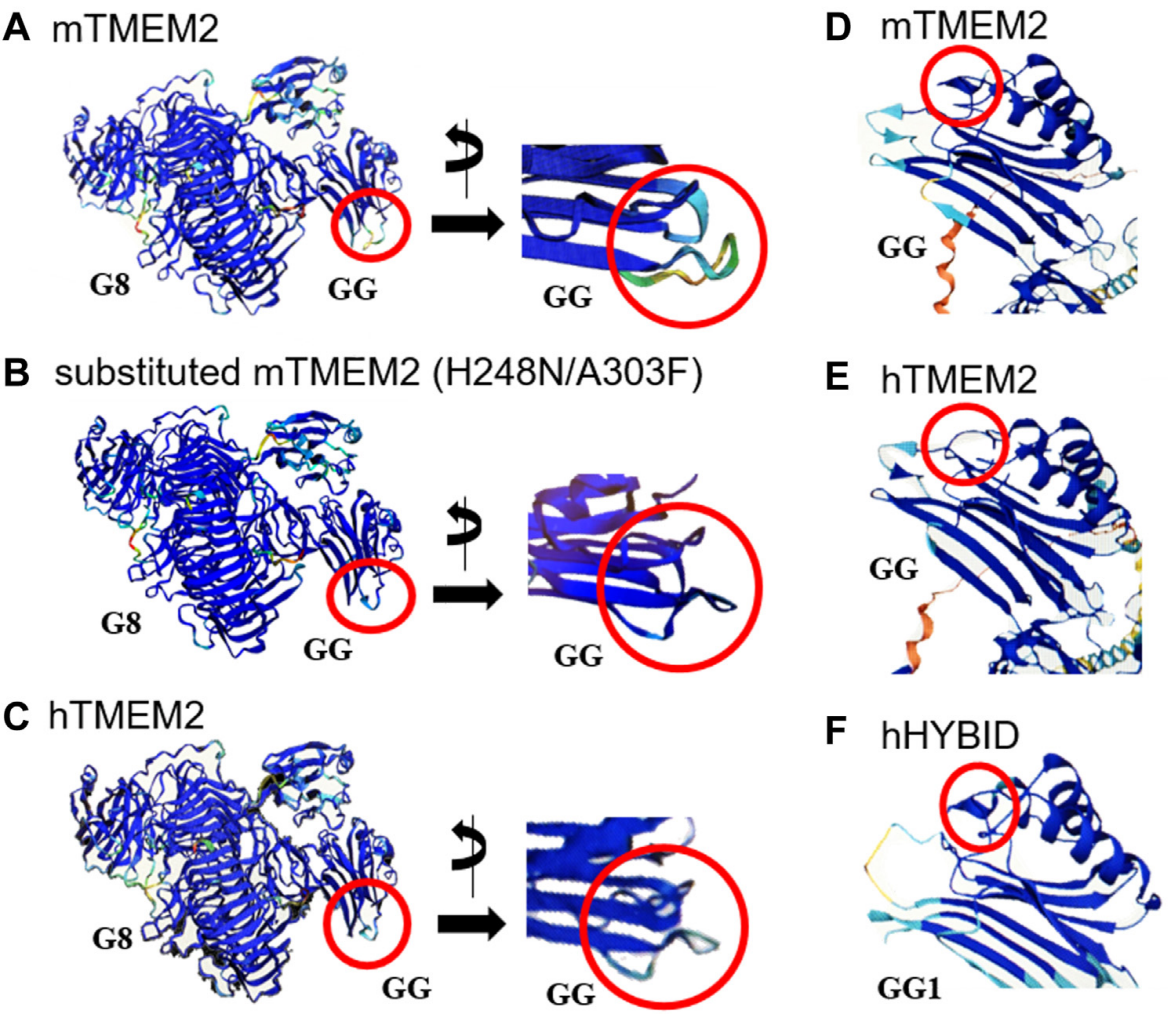
**Predicted 3D structures of mTMEM2, hTMEM2, and hHYBID.** The 3D structures of the TMEM2 extracellular domain (ECD) were predicted using the 3D structural analysis software AlphaFold2 (28). The GG domain structures of mTMEM2 (*A*), substituted mTMEM2 (H248N/A303F) (*B*), and hTMEM2 (*C*) were compared from two different orientations. The GG domain protrusions of each ECD with the different structures are shown in red circles. The 3D GG domain structures of mTMEM2 (*D*), hTMEM2 (*E*), and hHYBID (*F*) were obtained from AlphaFold data in UniProtKB (https://www.uniprot.org/help/uniprotkb) and compared. The GG domain protrusions of active mTMEM2 and hHYBID showed similar structures (*red circles*) but that of inactive hTMEM2 was different.

Using the 3D structural analysis software AlphaFold2 (28), the 3D structures of the mTMEM2 ECD, the substituted mTMEM2 ECD (H248N/A303F), and the hTMEM2 ECD were compared (Fig. 7, *A*–*C*). Comparison of the GG domain structure between active and inactive TMEM2 ECDs from two different orientations (Fig. 7, shown in red circles) indicates that the conformational change in the GG domain protrusion of the inactive mTMEM2 ECD (H248N/A303F) is similar to that of the hTMEM2 GG domain (Fig. 7, *B* and *C*). We also compared the GG domains of full-length mTMEM2 and hTMEM2 with the corresponding GG1 domain of the full-length hHYBID using AlphaFold in the UniProtKB database (https://www.uniprot.org/help/uniprotkb) (Fig. 7, *D*—*F*, shown in red circles). The conformational structure of inactive hTMEM2 (Fig. 7*E*) was different from those of active mTMEM2 and hHYBID, which have similar helix-like structures (Fig. 7, *D*, and *F*).

### The effects of proinflammatory cytokines and TGF-β in human dermal fibroblast are abrogated by the knockdown of TMEM2

**Figure 8:**
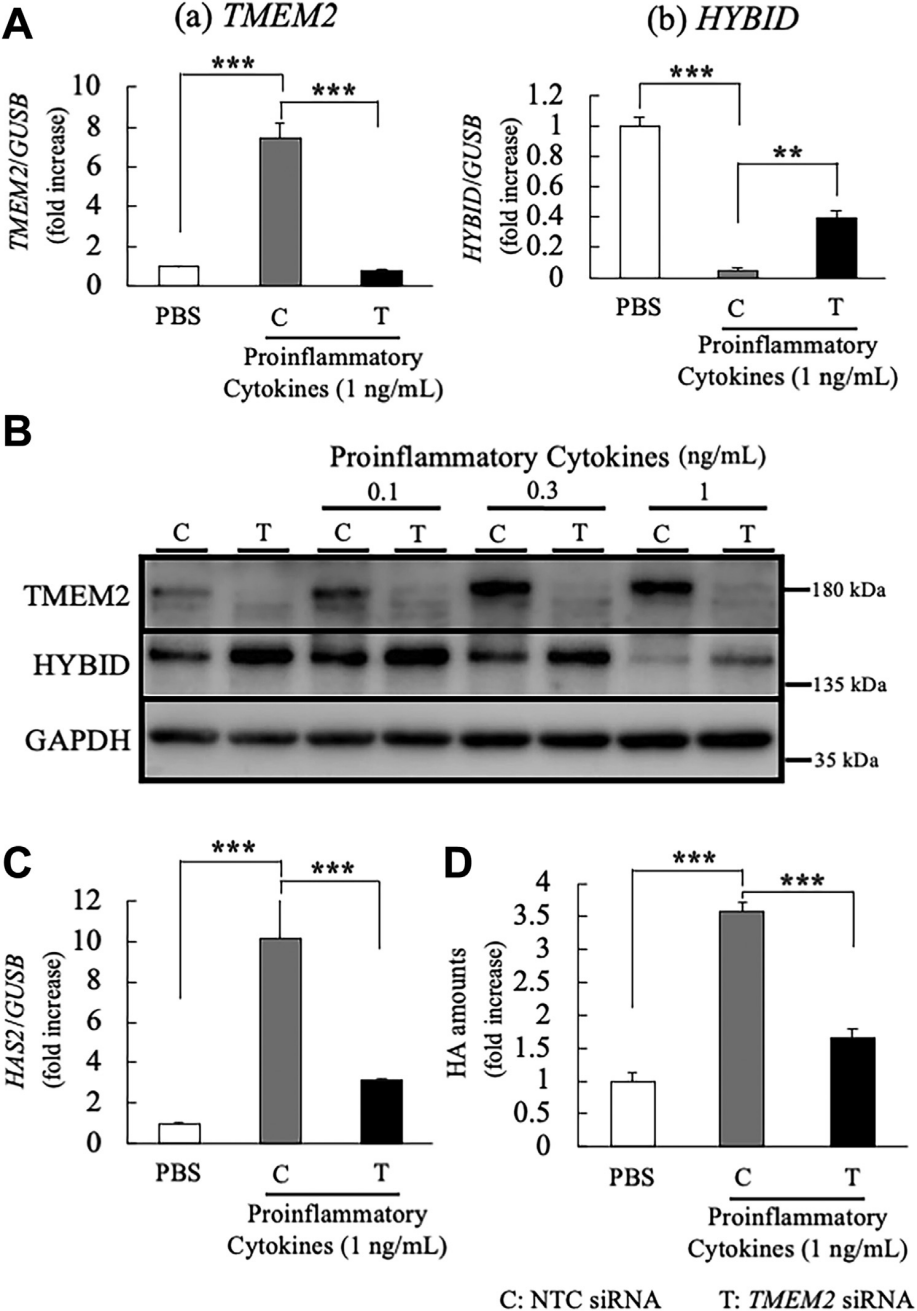
**The effects of proinflammatory cytokines in NHDFs are abrogated by the knockdown of hTMEM2**. *A*, the mRNA expression of human *TMEM2* and *HYBID* in proinflammatory cytokine-stimulated NHDFs. NHDF cells were pretreated with NTC (C) or *hTMEM2* (T) siRNAs. After 24 h, the media were changed with or without the proinflammatory cytokine cocktail (1.0 ng/ml PBS, same ratio mixture of TNFα, IL-1β, and IL-6). *TMEM2* and *HYBID* mRNA expression levels were determined by qRT-PCR after 24 h of stimulation. *GUSB* was used as a loading control. *B*, the expression levels of hHYBID and hTMEM2 proteins in NHDFs. After 48 h of stimulation with the cytokine cocktail (0.1, 0.3, and 1.0 ng/ml), the protein expression levels of hHYBID and hTMEM2 were determined *via* immunoblotting using specific antibodies. GAPDH was used as the loading control. *C*, the mRNA expression of human *HAS2* in cytokine-stimulated NHDFs. The total RNAs are the same as those used in *A*. The *HAS2* mRNA expression levels were determined by qRT-PCR. *GUSB* was used as a loading control. *D*, induction of HA in cytokine-stimulated NHDF culture medium. The concentration of HA in each medium was detected using an HA Quantification Kit. The culture medium was collected at the same time as (*B*). The control concentration (PBS) was set at 1.0 and the HA amount was shown as ratio (fold increase). *A*, *C*, and *D*, values represent the mean ± S.E.M. (n = 3). ∗∗*p* < 0.01, ∗∗∗*p* < 0.005 *versus* the control (PBS or C) (Tukey’s test).

The results described earlier demonstrate that hTMEM2 has no catalytic HA-degrading activity. Therefore, we examined the roles of hTMEM2 with respect to the regulatory function expected from our previous studies (13, 14). Twenty-four-hour stimulation of NHDFs with a cocktail of proinflammatory cytokines (containing 1 ng/ml of IL-1β, IL-6, and TNF-α) promoted the upregulation of *TMEM2* and *HAS2* mRNA expression levels (Fig. 8*A* (a), C,); however, *HYBID* mRNA expression was suppressed (Fig. 8*A* (b)). Notably, pretreatment with *TMEM2* siRNA canceled the effects of these proinflammatory cytokines (Fig. 8*A* (a), (b), and C).

The upregulation of hTMEM2 and downregulation of hHYBID by proinflammatory cytokines and the *TMEM2* siRNA-induced recovery of downregulated HYBID were confirmed at the protein level in a dose-dependent manner (Fig. 8*B*). Furthermore, changes in *HAS2* mRNA were correctly reflected in the amount of extracellular HA in the culture medium (Fig. 8*D*). These results indicate that the suppression of HYBID expression and enhancement of *HAS2* expression by the proinflammatory cytokines were canceled by *hTMEM2* silencing.

**Figure 9:**
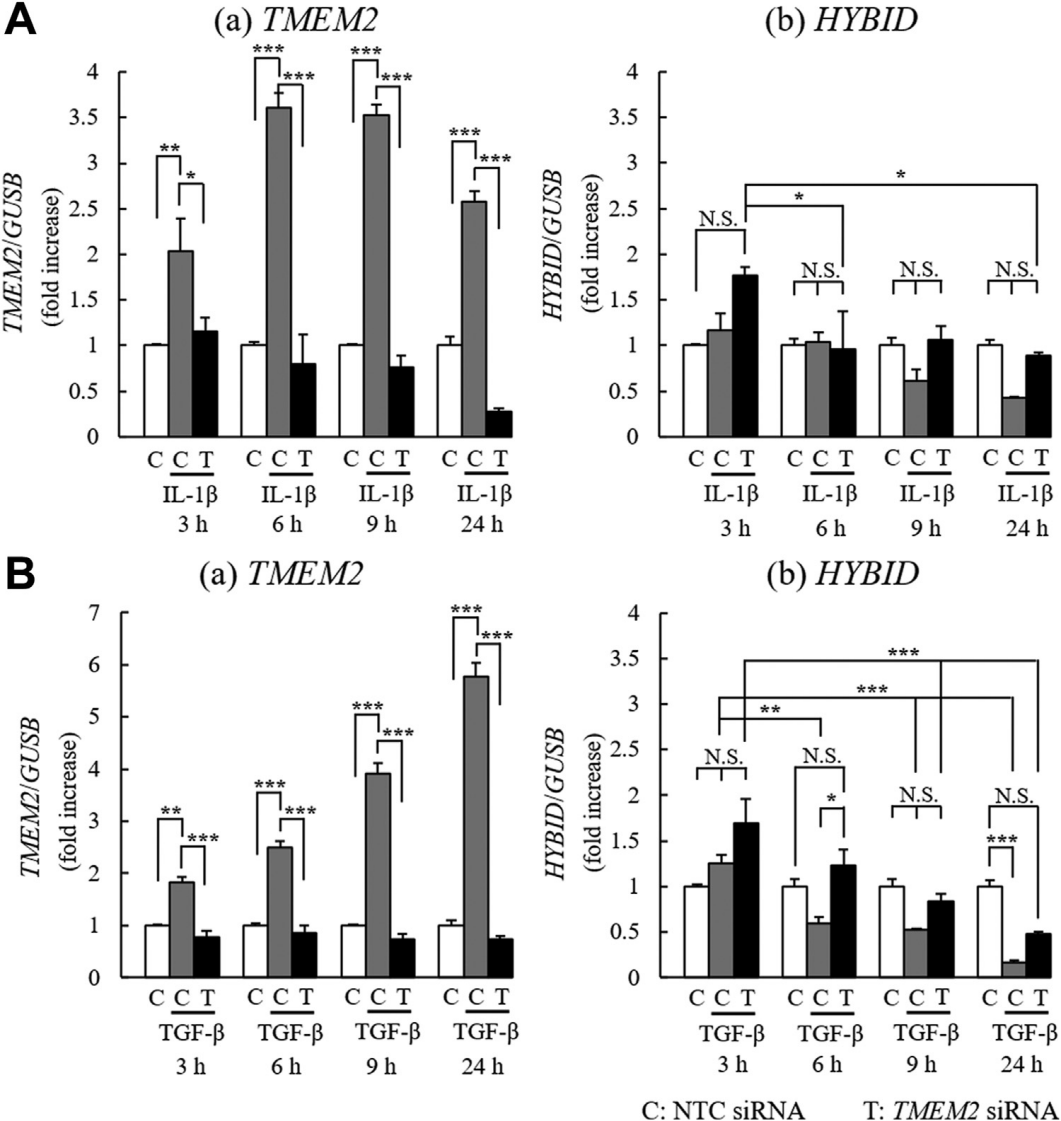
**IL-1β and TGF-β upregulate hTMEM2 and downregulate hHYBID, and hTMEM2 knockdown recovers these reactions in NHDFs**. NHDFs were pretreated with NTC (C) or *hTMEM2* (T) siRNA. *A*, time-dependent effect of IL-1β for mRNA expression of *hTMEM2* (*a*) and *hHYBID* (*b*). After 24 h of *hTMEM2* siRNA treatment, NHDFs were stimulated with or without IL-1β (1 ng/ml) for 3, 6, 9, and 24 h. *B*, time-dependent effect of TGF-β on mRNA expression of *hTMEM2* (*A*) and *hHYBID* (*B*). After 24 h of *TMEM2* siRNA treatment, cells were stimulated with or without TGF-β (10 ng/ml) for 3, 6, 9, and 24 h. The mRNA expression levels of *hTMEM2* and *hHYBID* were determined by qRT-PCR. *GUSB* was used as a loading control. Values represent the mean ± S.E.M. (n = 3). ∗ *p* < 0.05, ∗∗*p* < 0.01, ∗∗∗*p* < 0.005 *versus* the control (Tukey’s test). N.S., not significant.

To understand in detail the effects of hTMEM2-KD on the *HYBID* mRNA expression, we examined the time course of changes in the expression of these genes after IL-1β or TGF-β stimulation in hTMEM2-KD NHDFs (Fig. 9). When NHDFs were stimulated with IL-1β (1 ng/ml), *TMEM2* mRNA expression increased significantly from 3 h and reached a plateau at the 6 h time point (Fig. 9*A* (a), grey column). TGF-β (1 ng/ml) also induced *hTMEM2* mRNA expression significantly but gradually in a time-dependent manner (Fig. 9*B* (a), grey column). Both cytokine-induced increases in *hTMEM2* mRNA levels were completely eliminated by hTMEM2-KD, indicating that efficient KD by siRNA treatment was ensured during the experiment. In contrast, when NHDFs were stimulated with IL-1β or TGF-β, *HYBID* mRNA expression level decreased in a time-dependent manner, lagging behind and inversely proportional to the increase in *hTMEM2* expression (Fig. 9*A* (b) and Fig. 9*B* (b)), grey columns). In hTMEM2-KD NHDFs, the *HYBID* mRNA expression level remained unchanged 6 h to 24 h after IL-1β stimulation, and the suppressive effect of IL-1β on *HYBID* expression was recovered to the level of the non-stimulated control (Fig. 9*A* (b), open column *versus* black column). In TMEM2-KD NHDFs stimulated with TGF-β, the *HYBID* mRNA expression level (Fig. 9*B* (b), black column) was always higher than that of control (Fig. 9*B* (b), grey column) during the time course examined, although the suppressive effect of TMEM2-KD was partial at 24 h. The results shown in Figures 8 and 9 indicate that the inflammatory cytokines (such as IL-1β) and TGF-β upregulate hTMEM2 expression, and then suppress HYBID expression *via* hTMEM2 and that *HAS2* expression with increased HA production is also upregulated *via* hTMEM2.

## Discussion

Recently, mTMEM2 with high structural similarity to HYBID was proposed as a cell surface hyaluronidase (23). However, we previously found that the knockdown of hTMEM2, a human homolog of mTMEM2, unexpectedly promoted HA depolymerization by upregulating HYBID expression in NHDFs (13, 14). In the present study, we clarified that hTMEM2 is not a catalytic HA-degrading enzyme. Our results from the experiments testing the various Chimera proteins strongly suggest that the mGG domain in the mouse-derived C terminal sequence (including the PbH1 repeat) is essential for the HA-degrading activity of the protein. HYBID, which depolymerizes HA by clathrin-mediated endocytosis, shares a 48% amino acid sequence homology with the extracellular domain of TMEM2 and contains similar domains, such as the G8, GG, and PbH1 repeat. Recently, it has been reported that the G8 domain is also essential for the HA-depolymerizing activity of HYBID and may interact with several proteins, including annexin-1 (29). The Chimera 1 protein, which contained the hG8 domain instead of mG8, exhibited HA-degrading activity, indicating that mouse and human G8 domains are interchangeable for this function. Therefore, we focused on the differences in amino acid residues between hTMEM2 and HA-degrading mTMEM2, hHYBID, and mHYBID, as shown in Figure 5.

GG domains have been identified in members of the eukaryotic FAM3 superfamily (FAM3A, FAM3B, FAM3C, and FAM3D), POMGnT1 (protein O-linked mannose β-1,2-N-acetylglucosaminyl-transferase), phage gp35 proteins, and TMEM2 (30). Their roles remain to be fully elucidated; however, it is possible that the GG domain of TMEM2 contributes to HA-degrading activity, since it has been reported that substitution of amino acid residues such as R187C and R187H in the GG1 domain of hHYBID (12), and R265C, D273N and D286N in the GG domain of mTMEM2 (23), caused loss of HA-degrading activity. In this study, we found that the simultaneous substitution of two mTMEM2 residues to the corresponding amino acids of inactive hTMEM2 (H248N and A303F) resulted in a loss of HA-degrading activity (Fig. 6*D*). Together these findings suggest that the GG domain of TMEM2 and the corresponding GG1 domain of HYBID play essential roles in the HA-degrading activity of these proteins.

The 3D structure in the GG domain protrusion of the inactive mTMEM2 ECD (H248N/A303F) was similar to that of the hTMEM2 GG domain, and the conformational structure of inactive full-length hTMEM2 was different from those of active mTMEM2 and hHYBID which have similar helix-like structures (Fig. 7). This strongly suggests that this projection of the GG domain is essential for the HA-degrading activity of HYBID and TMEM2 and also might be involved in their molecular interactions with the HA substrate.

Our data indicate that hTMEM2 lacks HA-degrading activity but regulates HA metabolism by downregulating HYBID and upregulating HAS2 gene expression to promote the accumulation of HMW-HA. Furthermore, we showed that IL-1β and TGF-β regulate HA metabolism *via* hTMEM2 expression (Figs. 8 and 9). Comparative analysis of the 3D structures of the intracellular domain (ICD) of hTMEM2 and mTMEM2 showed, interestingly, the existence of a clear α-helix structure only in the hTMEM2 ICD (Fig. S1, *A* and *B*). In addition, a proline-rich sequence (PPPPPP) as a binding site for the SH3 domain (31) and a nuclear migration motif (KQKRHK) (32) were found in the central region of the hTMEM2 ICD and at the C-terminus, respectively. These findings suggest that the hTMEM2 ICD may regulate intracellular signaling pathways by interacting with tyrosine kinases with SH3 domains (33) or may regulate a pathway of genes including HYBID and HAS2. In addition, it is possible that hTMEM2 regulates the enzymatic activity of HYBID and/or HAS2 by post-translational modulation, such as O-GlcNAcylation of HAS2 (34) and the binding of annexin-1 to the G8 domain of HYBID (29). Further studies are needed to elucidate such a possibility.

It is unclear whether the mTMEM2 ICD has a regulatory function in gene expression. Tobisawa *et al*. established tamoxifen-induced mTMEM2 knockout mice (Tmem2iKO) (24), in which the ECD was conditionally knocked out but the ICD remained. Therefore, the regulatory function of ICD cannot be evaluated in Tmem2iKO mice. *mTMEM2* is highly expressed and dominant in many tissues in mice, whereas *mHYBID* is expressed at the same level as *mTMEM2* in only the brain, spleen, and synovium (23). This may indicate that mTMEM2 functions as a dominant hyaluronidase rather than as a regulator in mice, although our studies using mHYBID-KO mice have confirmed that mHYBID also contributes to HA depolymerization in the central nervous system and cartilage junctions of mice (22, 35). Further study is needed to elucidate the regulatory function of mTMEM2 ICD using mouse cells expressing both *mHYBID* and *mTMEM2*.

Despite the high sequence homology between human and mouse TMEM2, their physiological functions are unexpectedly oppositional. Since the discovery of mTMEM2 as a hyaluronidase, there have been many reports on mouse and human TMEM2 (24, 25, 26, 27, 36, 37). In particular, studies on hTMEM2 have been reported with the assumption that it is a catalytic hyaluronidase, but no reports have directly evaluated its HA-degrading activity. For example, Schinzel *et al*. used human fibroblasts in which hTMEM2 was knocked out by CRISPR/Cas9 to evaluate tunicamycin-induced ER stress sensitivity (26). They showed that hTMEM2-KO cells displayed increased sensitivity to ER stress, whereas cells overexpressing hTMEM2 had improved cell survival. The authors thus speculated that hTMEM2 degrades HA into LMW- HA, which is necessary and sufficient to improve cell survival. However, we can explain that overexpressed hTMEM2 upregulates HAS2 and downregulates HYBID to accumulate HMW-HA, which possibly improves cell survival and longevity (38). The lifespan extension of *C. elegans* by hTMEM2 overexpression may be due to the effects of hTMEM2-regulated gene expression, including HAS2 and HYBID, rather than hyaluronidase activity. Irie *et al*. also reported that hTMEM2 regulates cell adhesion and migration *via* HA degradation of cell focal adhesion sites using a variety of human-derived tumor cell lines (27). In this case, phenotypic recovery was evaluated for cells with hTMEM2 knockdown by introducing mTMEM2 or by adding small HA fragments. Further studies to analyze hTMEM2-KO-induced changes in other gene expressions are needed to elucidate the mechanism for the effects of hTMEM2 on tumor adhesion and migration. In several tumors, HA degradation was shown to be involved in invasion and metastasis since HMW-HA inhibits cell motility and tumor cell proliferation (39, 40), whereas LMW-HA is associated with cell migration and invasion (41, 42). Notably, it was reported that hTMEM2 expression was downregulated in bladder cancer invasion, in which TMEM2 expression was higher in non-invasive cancers, whereas invasive cancer cells were less likely to express hTMEM2 during muscle invasion (37).

In summary, we have shown that hTMEM2, unlike mTMEM2, has no catalytic HA-degrading activity and is a regulator that accumulates HMW-HA through the regulation of HYBID and HAS2 expression. The lack of enzymatic activity by hTMEM2 is caused by changes in at least two amino acid residues (H248/A303) in the GG domain of active mTMEM2. Our findings need to be further extrapolated regarding the roles of hTMEM2 in diseases such as cancer, arthritis, and hearing loss, and also in ECM-related cell functions. Moreover, we have provided enzymatic insights into the structures required for the HA-degrading activity of HYBID and mTMEM2. Future studies are needed to elucidate the regulatory mechanism of gene expression focusing on the ICD shedding, and the intracellular domains of hTMEM2.

## Experimental procedures

### Cell cultures and stimulation

Human embryonic kidney 293 (HEK293) (KAC Co, Ltd (Kyoto, Japan)) and HEK293T cells were cultured in Eagle’s minimum essential medium (FUJIFILM Wako Pure Chemical, Osaka, Japan) supplemented with 1 × nonessential amino acids (FUJIFILM Wako Pure Chemical) and 10% (v/v) fetal bovine serum (FBS). HEK293 cells were seeded at a density of 7.5 × 10^5^ cells/well in a 24-well plate and incubated with or without IL-1β (1 ng/ml) and TGF-β1 (10 ng/ml) (FUJIFILM Wako Pure Chemical).

Normal human dermal fibroblasts (NHDFs), Detroit 551, were obtained from the American Type Culture Collection (VA, USA). The Detroit 551 cells were maintained in Eagle’s minimum essential medium supplemented with 1 × nonessential amino acids, 1 mM sodium pyruvate, and 10% (v/v) FBS. The Detroit 551 cells were seeded at a density of 2.5 × 10^4^ cells/well in a 24-well plate and incubated with or without a mixture of the cytokines (0.1–1.0 ng/ml, TNF-α, IL-1β, and IL-6), IL-1β (1.0 ng/ml) or TGF-β (10 ng/ml) (FUJIFILM Wako Pure Chemical).

### Plasmids

Plasmids harboring human and mouse *TMEM2* open reading frames were purchased from ORIGENE (RC224793) and Promega (ORK00828), respectively. The cDNAs for TMEM2 were amplified by PCR using PrimeSTAR Max DNA polymerase (Takara Bio) and primer pairs #1, #2 and #3, and #4, listed in Table S1. These primers were designed to include restriction enzyme sites and the FLAG epitope coding sequence. The amplified cDNA fragments were inserted into pcDNA3.1 (Invitrogen, CA, USA), resulting in *hTMEM2*-FLAG-pcDNA3.1 and *mTMEM2*-FLAG-pcDNA3.1. Using these wild-type *TMEM2* expression plasmids as templates, recombinant PCR was performed to make chimera and point mutation constructs. Plasmids were purified by HiSpeed Plasmid Midi Kit (QIAGEN, Venlo, Netherlands). Schematic diagrams of the recombinant PCR and the primers are shown in Fig. S2, and Tables S1 and S2.

### Plasmid DNA and siRNA transfection

The HEK293 or HEK293-T cells were seeded at 7.5 × 10^5^ cells/well in a 24-well plate. Each concentration (0.125–1.0 μg) of expression plasmids was mixed with 0.75 to 3.0 μl Lipofectamine 2000 Transfection Reagent (Thermo Fisher Scientific, MA, USA) in 100 μl Opti-MEM (Thermo Fisher Scientific). After 8 h of transfection, the medium was changed to 10% (v/v) FBS culture medium.

Detroit 551 cells were seeded at 3.0 × 10^４^ cells/well in a 24-well plate. After 24 h, a final concentration of 2 nM Stealth RNAi siRNA (Invitrogen) was transfected into the cells with Lipofectamine RNAiMAX Reagent (Thermo Fisher Scientific) using Opti-MEM (Thermo Fisher Scientific). The siRNA oligonucleotide sequences were as follows: *TMEM2* siRNA: 5′-CAGGAUGCUGGAAUAUGGUAUUUAU-3′ (sense), 5′-AUAAAUACCAUAUUCUCGCAUCCUG-3′ (antisense). Negative control (NTC) siRNA (Thermo Scientific) was used as a negative control.

### Quantitative reverse transcription PCR

Total RNA was extracted using a NucleoSpin RNA kit (Takara Bio). Complementary DNA was prepared using PrimeScript RT Master Mix enzyme (Takara Bio) in a reaction conducted at 37 °C for 15 min. PCR amplification was conducted in the same method as described in our previous reports (13, 14); running 45 cycles under the following conditions: denaturation for 5 s at 95 °C; annealing and extension for 30 s at 60 °C. Glucuronidase beta (*GUSB*) was used as the reference gene for normalization.

The PCR primer sequences were:

*HYBID*-forward: 5′-GGCTTCTGAGCCGGAACATC-3′

*HYBID*-reverse: 5′-GCTGCCTTAAATCCCAGAGCAA-3′

*TMEM2*-forward: 5′-TCCACAGTACCAGCCTGTCGTC-3′

*TMEM2*-reverse: 5′-TGATGGATAGCAAAGGCCAACTC-3′

*HAS2*-forward: 5′-CCTTCAGAGCACTGGGACGA-3′

*HAS2*-reverse: 5′-AGATGAGGCTGGGTCAAGCATAG-3′

*GUSB*-forward: 5′-CATTATTCAGAGCGAGTATGGAGCA-3′

*GUSB*-reverse: 5′-TCTTCAGTGAACATCAGAGGTGGA-3′

### Immunoblotting analysis

Total cell extract was prepared with 1 × RIPA buffer [50 mmol/L-Tris-HCl buffer (pH 7.6), 150 mmol/L-NaCl, 1 %-Nonidet P40 substitute (w/v), 0.5 %-sodium deoxycholate (w/v), protease inhibitor cocktail, 0.1 %-SDS (w/v)] (FUJIFILM Wako Pure Chemical). The samples were separated on 10% SuperSep Ace gels (Wako) and transferred to PVDF membranes (Merck). Nonspecific binding was blocked at room temperature (15–25 °C) for 15 min with Blocking One-P (Nacalai Tesque, Kyoto, Japan). The membranes were incubated overnight at 4 °C with the following antibodies: anti-KIAA1199 (HYBID) rabbit polyclonal antibody (pAb) (1:1000) (Sigma Aldrich, MO, USA), anti-TMEM2 rabbit pAb (1:500) (Sigma Aldrich), anti-FLAG M2 mouse mAb (1:2000) (Sigma Aldrich) and anti-GAPDH mouse mAb (1:2000) (Santa Cruz Biotechnology). The primary antibodies were detected using HRP-conjugated secondary antibodies (GE Healthcare). Bands were detected *via* ImmunoStar LD (FUJIFILM Wako Pure Chemical) using an Amersham Imager 680 (GE Healthcare Bioscience, NJ, USA).

### HA depolymerization assay using gel permeation chromatography analysis

A final concentration of 10 μg/ml FITC-labeled HMW-HA (FA-HA H2; PG Research, Tokyo, Japan) (1200–1600 kDa) was added to the culture medium. After 24 or 48 h of culture, the culture medium was collected and applied to a Sepharose CL-2B (GE Healthcare Bioscience) column (0.7 × 50 cm) equilibrated with 0.5 M NaCl in distilled water. The flow rate was 0.15 ml/min, and 0.3 ml fractions were collected. The fluorescence of each fraction was measured using a GloMax-Multi Detection System (E x .490 nm/Em.510–570 nm, Promega).

### Quantification of HA in the culture medium

The concentration of HA in cell culture media was determined using an HA Quantification Kit (PG Research) according to the manufacturer’s protocol.

### Statistical analysis

All the data are expressed as the mean ± standard error of the mean (S.E.M.). Statistical comparisons were made by Tukey’s test. *p* values < 0.05 were considered statistically significant. All statistical analyses were performed using the SPSS software program (ver. 24.0, IBM, IL, USA).

## Data availability

The data that support the findings of this study are available from the corresponding author [inoshin@gifu-pu.ac.jp] upon reasonable request.

## Supporting information

This article contains supporting information.

## Conflict of interest

The authors have no conflicts of interest to declare. The authors belong to a laboratory (Cosmetic Health Science) supported by Ichimaru Pharcos Co, Ltd.
