# Supplementary material for: Human TMEM2 is not a catalytic hyaluronidase, but a regulator of hyaluronan metabolism via HYBID (KIAA1199/CEMIP) and HAS2 expression
Source: J Biol Chem. 2023 May 16;299(6):104826. doi: 10.1016/j.jbc.2023.104826 (PMC10276149; doi:10.1016/j.jbc.2023.104826)
Supplement: Supporting information [file mmc1.pdf]

Fig. S1

A hTMEM2

1 MYATDSRGHS PAFLQPQNGN SRHPSGYVPG KVV**PLRPPPP** 40
41 **P**KSQASAKFT SIRREDRATF AFSPEEQQAQ RESQ**KQKRHK** 80
81 NT 82

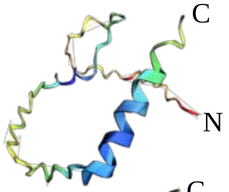

B mTMEM2

1 MYAAGSRGHS PAFLQPQNGN GHRSPGYVPG KVV**PLRPAPP** 40
41 **P**KNHASAKLT SRSQDAPATF AFSPEEQRTP SES**RKRKRHK** 80
81 NT 82

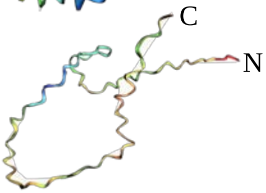

Figure S1. The 3D structures of hTMEM2 and mTMEM2 intracellular domains (ICD) predicted using AlphaFold2 and each amino acid sequences (A and B, respectively) were compared. The α-helix structure appeared only in the hTMEM2 ICD (A). The proline-rich sequence (PPPPPP, underlined) (a binding site for the SH3 domain) and a nuclear migration motif (KQKRHK) are shown in the center of the ICD and the C-terminus (red).

Fig. S2

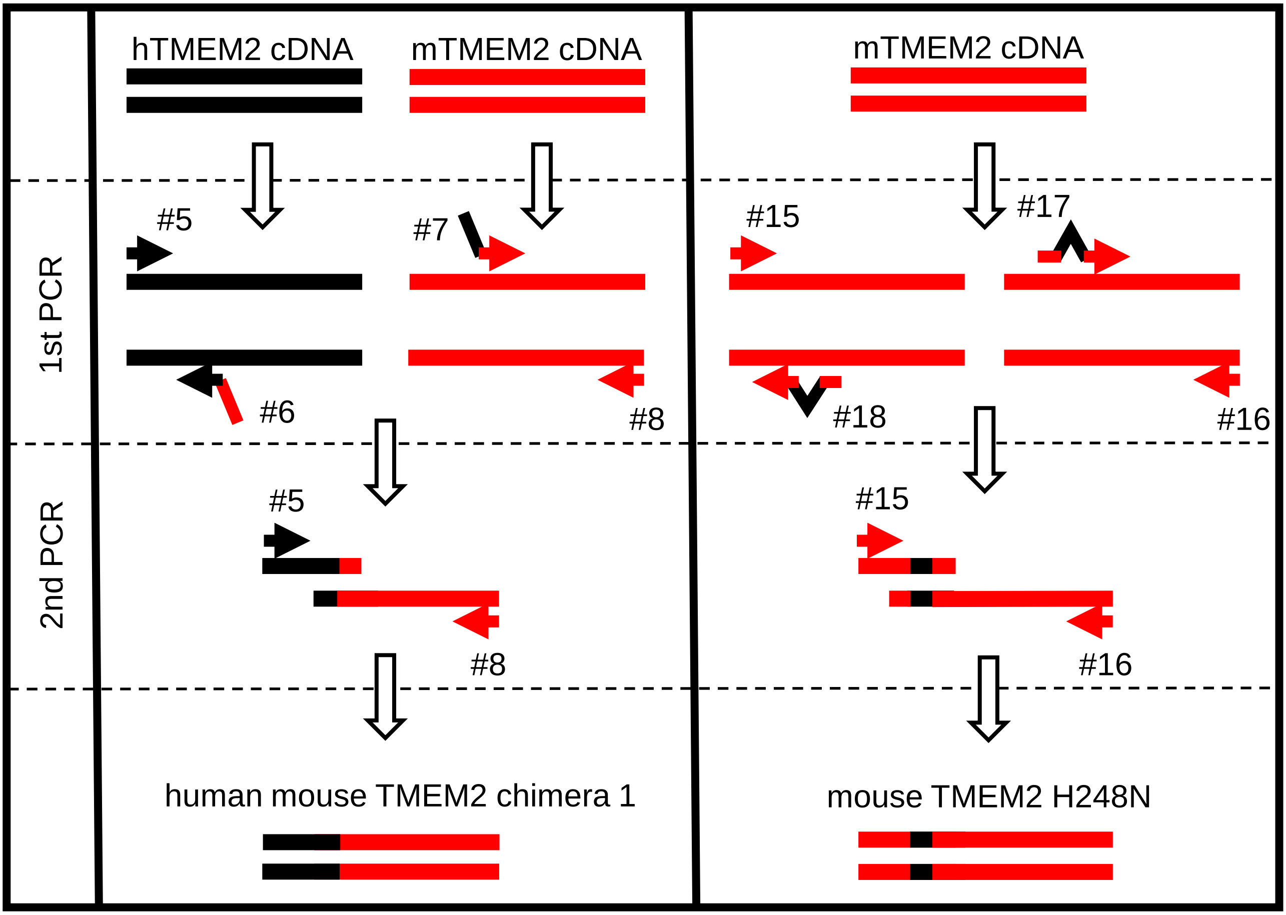

Figure S2. Schematic representation of the recombinant PCR. Two examples are shown.

Left: To make human and mouse TMEM2 chimera 1, 1st PCR was performed using (1) primers #5, and #6 with a template hTMEM2-FLAG-pcDNA3.1 and (2) primers #7, and #8 with a template mTMEM2-FLAG-pcDNA3.1. In the 2nd PCR, resulting two fragments in the 1st PCR were mixed and connected using primers #5 and #8.

Right: To make mTMEM2 H248N, 1st PCR was performed using (1) primers #15, #18 and (2) primers #16, and #17 with a template mTMEM2-FLAG-pcDNA3.1. In the 2nd PCR, the resulting two fragments in 1st PCR was mixed and connected by PCR using primers #15 and #16.

After this recombinant PCR, The final PCR products were inserted into the restriction enzyme-digested pcDNA3.1 by using standard subcloning procedures. Plasmid sequences were confirmed by via Sanger sequencing.

Table S1

Table S1 Primer sequences used in this study

Primer Number	Primer name	Primer sequence (5' to 3')
#1	NheI-*hTMEM2*-ATG	ATAGCTAGCGCCACCATGTATGCCACTGATTCCAGGG
#2	XhoI-*hTMEM2*-FLAG-Stop	GGCCTCGAGTTACTTGTCGTCATCGTCTTTGTAGTCATGTGCTTTTGAAGCTTGCTTT
#3	SpeI-*mTMEM2*-ATG	ATAACTAGTGCCACCatgtatgccgctggttccag
#4	XhoI-*mTMEM2*-FLAG-Stop-XhoI	GGCCTCGAGTTACTTGTCGTCATCGTCTTTGTAGTCaagcactttcaaagcctgctgt
#5	KpnI-*hTMEM2*-ATG	TTAGGTACCGCCACCATGTATGCCACTGATTCCAG
#6	*hmTMEM2*-chimera-R1	atgtcctctGTGCCCCATGTAACTCCAGTG
#7	*hmTMEM2*-chimera-F1	ACATGGGGCacagaggacatcatggacgatg
#8	bGH-polyA-Rev	CAACAGATGGCTGGCAACTA
#9	*hmTMEM2*-chimera-R2	ttccttgacTCCATTCACTATAAGCTGTCAC
#10	*hmTMEM2*-chimera-F2	TAGTGAATGgagtcaaggaatctccctctc
#11	*hmTMEM2*-chimera-R3	CGCCTTCAAtccattcactgtaggctgtcac
#12	*hmTMEM2*-chimera-F3	cagtgaatgGATTGAAGGCGTTTCTCTTTC
#13	*hmTMEM2*-chimera-R4	ATGCCTTCCgtgccccgtgtaactccagtg
#14	*hmTMEM2*-chimera-F4	acacggggcACGGAAGGCATCGTGGACGTTG
#15	KpnI-*mTMEM2*-ATG	TTAGGTACCGCCACCatgtatgccgctggttccag
#16	*mTMEM2*-BbvC1-rev	catgaatcctccatctctcc
#17	*mTMEM2*-H248N-F	gaggactctgAATtcttcagg
#18	*mTMEM2*-H248N-R	cctgaagaATTcagagtcctc
#19	*mTMEM2*-V279I-F	cacagccagaATTttagaaaa
#20	*mTMEM2*-V279I-R	ttttctaaAATtctggctgtg
#21	*mTMEM2*-A303F-F	agtttttgagaTTCcaggagcccgg
#22	*mTMEM2*-A303F-R	ccgggctcctgGAAtctcaaaaact
#23	*mTMEM2*-K337E-F	gttggggagtGAActgatcca
#24	*mTMEM2*-K337E-R	tggatcagTTCactccccaac
#25	*mTMEM2*-S359T-F	tggtggaagtACTtcctgcaa
#26	*mTMEM2*-S359T-R	ttgcaggaagTACttccacca

Note: Restriction enzyme sites and FLAG epitope coding sequence are single and double underlined, respectively. Initiation and stop codons are in bold. *mTMEM2* sequences are shown in lowercase.

Table S2

Table S2 Primers and templates for the construction of chimera and single or double substitutes *TMEM2* by recombinant PCR

Plasmid Number	Plasmid name	PCR1		PCR2		PCR3		Description
		Template	Primer pair	Template	Primer pair	Template	Primer pair	
[3]	*hmTMEM2* chimera 1-FLAG-pcDNA3.1	[1]	#5, #6	[2]	#7, #8	PCR 1&2 mixture	#5, #8	hTMEM2 (1-235) - mTMEM2 (236-1383)
[4]	*hmTMEM2* chimera 2-FLAG-pcDNA3.1	[1]	#5, #9	[2]	#10, #8	PCR 1&2 mixture	#5, #8	hTMEM2 (1-399) - mTMEM2 (400-1383)
[5]	*hmTMEM2* chimera 3-FLAG-pcDNA3.1	[2]	#3, #11	[1]	#12, #8	PCR 1&2 mixture	#3, #8	mTMEM2 (1-399) - hTMEM2 (400-1383)
[6]	*hmTMEM2* chimera 4-FLAG-pcDNA3.1	[2]	#3, #13	[1]	#14, #8	PCR 1&2 mixture	#3, #8	mTMEM2 (1-235) - hTMEM2 (236-1383)
[7]	*mTMEM2* H248N-FLAG-pcDNA3.1	[2]	#15, #18	[2]	#17, #16	PCR 1&2 mixture	#15, #16	mTMEM2 H248N
[8]	*mTMEM2* V279I-FLAG-pcDNA3.1	[2]	#15, #20	[2]	#19, #16	PCR 1&2 mixture	#15, #16	mTMEM2 V279I
[9]	*mTMEM2* A303F-FLAG-pcDNA3.1	[2]	#15, #22	[2]	#21, #16	PCR 1&2 mixture	#15, #16	mTMEM2 A303F
[10]	*mTMEM2* K337E-FLAG-pcDNA3.1	[2]	#15, #24	[2]	#23, #16	PCR 1&2 mixture	#15, #16	mTMEM2 K337E
[11]	*mTMEM2* S359T-FLAG-pcDNA3.1	[2]	#15, #26	[2]	#25, #16	PCR 1&2 mixture	#15, #16	mTMEM2 S359T
[12]	*mTMEM2* double substitute -FLAG-pcDNA3.1	[7]	#15, #22	[7]	#21, #16	PCR 1&2 mixture	#15, #16	mTMEM2 H248N/A303F

Note: Plasmid [1] is *hTMEM2*-FLAG-pcDNA3.1 that express wild-type *hTMEM2* tagged with FLAG epitope. Plasmid [2] is *mTMEM2*-FLAG-pcDNA3.1 that express wild-type *mTMEM2* tagged with FLAG epitope. See *Experimental procedures* in the main text for the construction of these plasmids.
